# Metagenome-enabled models improve genomic predictive ability and identification of herbivory-limiting genes in sweetpotato

**DOI:** 10.1093/hr/uhae135

**Published:** 2024-05-10

**Authors:** Alhagie K Cham, Alison K Adams, Phillip A Wadl, Ma del Carmen Ojeda-Zacarías, William B Rutter, D Michael Jackson, D Dewayne Shoemaker, G Craig Yencho, Bode A Olukolu

**Affiliations:** Department of Entomology and Plant Pathology, University of Tennessee, Knoxville, TN 37996, USA; Department of Entomology and Plant Pathology, University of Tennessee, Knoxville, TN 37996, USA; Genome Science and Technology, University of Tennessee, Knoxville, TN 37916, USA; Department of Plant Pathology, University of Georgia, Griffin, GA 30223, USA; US Vegetable Laboratory, United States Department of Agriculture, Agriculture Research Service, Charleston, SC 29414, USA; Faculty of Agronomy, Autonomous University of Nuevo León, Francisco Villa s/n, Col. Ex Hacienda El Canadá, 66050, General Escobedo, Nuevo León, México; US Vegetable Laboratory, United States Department of Agriculture, Agriculture Research Service, Charleston, SC 29414, USA; US Vegetable Laboratory, United States Department of Agriculture, Agriculture Research Service, Charleston, SC 29414, USA; Department of Entomology and Plant Pathology, University of Tennessee, Knoxville, TN 37996, USA; Department of Horticultural Science, North Carolina State University, Raleigh, NC 27695, USA; Department of Entomology and Plant Pathology, University of Tennessee, Knoxville, TN 37996, USA; Genome Science and Technology, University of Tennessee, Knoxville, TN 37916, USA

## Abstract

Plant–insect interactions are often influenced by host- or insect-associated metagenomic community members. The relative abundance of insects and the microbes that modulate their interactions were obtained from sweetpotato (*Ipomoea batatas*) leaf-associated metagenomes using quantitative reduced representation sequencing and strain/species-level profiling with the Qmatey software. Positive correlations were found between whitefly (*Bemisia tabaci*) and its endosymbionts (*Candidatus Hamiltonella defensa*, *Candidatus Portiera aleyrodidarum*, and *Rickettsia* spp*.*) and negative correlations with nitrogen-fixing bacteria that implicate nitric oxide in sweetpotato–whitefly interaction. Genome-wide associations using 252 975 dosage-based markers, and metagenomes as a covariate to reduce false positive rates, implicated ethylene and cell wall modification in sweetpotato–whitefly interaction. The predictive abilities (PA) for whitefly and *Ocypus olens* abundance were high in both populations (68%–69% and 33.3%–35.8%, respectively) and 69.9% for *Frankliniella occidentalis*. The metagBLUP (gBLUP) prediction model, which fits the background metagenome-based Cao dissimilarity matrix instead of the marker-based relationship matrix (G-matrix), revealed moderate PA (35.3%–49.1%) except for *O. olens* (3%–10.1%). A significant gain in PA after modeling the metagenome as a covariate (gGBLUP, ≤11%) confirms quantification accuracy and that the metagenome modulates phenotypic expression and might account for the missing heritability problem. Significant gains in PA were also revealed after fitting allele dosage (≤17.4%) and dominance effects (≤4.6%). Pseudo-diploidized genotype data underperformed for dominance models. Including segregation-distorted loci (SDL) increased PA by 6%–17.1%, suggesting that traits associated with fitness cost might benefit from the inclusion of SDL. Our findings confirm the holobiont theory of host–metagenome co-evolution and underscore its potential for breeding within the context of G × G × E interactions.

## Introduction

Sweetpotato (*Ipomoea batatas*) plays a significant role in addressing global hunger and malnutrition; however, the impact of major pests, such as whiteflies (*Bemisia tabaci*) and sweetpotato weevils (*Cylas* spp*.*), is still a significant concern for farmers [1]. While whiteflies do not directly target the harvestable part (storage roots) of the crop like weevils do, they feed on phloem sap, consequently reducing plant growth rate and yield [2]. Additionally, pathogen transmission that negatively affects yield often occurs through wounding sites [3]. The investigation of the metagenome is important for understanding multipartite and multitrophic interactions that directly or indirectly modulate host phenotypic expression. Beneficial microbes within the host-associated metagenome can enhance plant growth and protect plants from pathogens and pests. Earlier studies have highlighted the host-adapted metagenome as a potential source of beneficial organisms [4]. Identifying and/or selecting alleles that drive the recruitment of these beneficial microbes can expand the repertoire of the defense response, in addition to host resistance.

Investigating multiway interactions within metagenomic communities provides insights into how plant defense pathways are modulated. While high population pressure from foliar pests can cause direct damage to plants and crop yield, low population pressure can result in yield loss when the pests transmit viral pathogens. In severe cases, viral infections transmitted by these pests can result in yield losses ranging from 30% to 50% and, in some instances, total yield losses of up to 99% [5, 6]. These viruses can consequently be transmitted vertically through planting materials, seeds, and pollen [7]. These viruses have also demonstrated the ability to predispose plants to fungal and bacterial infections that contribute to a further reduction in productivity. Of particular concern are insect vectors, such as whiteflies, thrips, and aphids, that feed on the phloem and mediate a diverse array of plant–viral pathosystems [8].

Herbivorous insects have co-evolved with their plant hosts, developed effectors that manipulate the host defense response, and altered resource allocation patterns to their advantage. On the contrary, when attacked by herbivores, the plant host undergoes changes in resource allocation, such as compensatory growth responses, which can impact other organisms that obtain nutrition from the plant [9]. The plant has evolved to recognize pathogens and trigger a defense response against the invading organisms. Plant defense response to herbivory involves dynamic and intricate systems, including hypersensitive response (HR), production of toxic chemicals and volatiles, establishment of structural barriers, and the attraction of predators. While the mechanisms underlying HR and its physiological and ultrastructural consequences are often assumed to be highly conserved across higher plants and typically associated with viral, bacteria, fungal, and oomycetes pathogens, HR can also be triggered by insects [10]. The ethylene and jasmonic acid (ET–JA) signaling pathway plays a pivotal role in mediating induced plant defenses [11]. This crosstalk between ET-JA pathway can give rise to the synthesis of volatile organic compounds (VOCs) that function as airborne signals. These VOCs play a crucial role in deterring herbivores, either by attracting their predators or directly repelling them [12].

While an understanding of the genetic and mechanistic basis for host resistance can facilitate solutions targeted toward crop protection, breeding programs often depend on predictive models for crop improvement. Traditional breeding methods face limitations in achieving rapid breeding for polygenic traits and the complex genetics of polyploid crops. By accounting for allele dosage in polyploids, genomic prediction can accurately and rapidly enhance selection and advance genotypes with favorable alleles [13]. Genomic selection can expedite the process by pinpointing individuals with higher rates of genetic gain for specific traits. Factors that can enhance genomic prediction accuracy include incorporating dominance effects across various dosage models, selecting the best genomic model, and considering factors such as population structure, microbiome modulation of phenotypic expression, sample size, genetic architecture, and the number of markers used [14]. The implementation of genomic selection, a form of marker-assisted selection, is often restricted by the host genotype, microbiome, and environment interactions (G_H_ × G_M_ × E), i.e. the change in genotype response to a change in metagenomic community composition and environment. Studies have accounted for the latter two components during genomic prediction by modeling the metagenome/microbiome and environment as covariates [15].

Recent advancements in data acquisition and sequencing technologies offer unprecedented opportunities for investigating complex traits and genes that control broad-spectrum and durable resistance. While low- and high-throughput phenotyping of plant diseases can be informative, they have inherent limitations, particularly in diseases that are asymptomatic or where symptoms are highly variable and influenced by abiotic and other biotic factors. Earlier studies have deployed phenotype-free assays to assess the abundance of pathogens and pests using molecular techniques such as quantitative or real-time polymerase chain reaction (qPCR), enzyme-linked immunosorbent assay, and loop-mediated isothermal amplification assay [16]. Although these quantitative assays are often straightforward, sometimes cost-effective, and target the pathogen of interest, the pathogen abundance is not always correlated with disease symptoms since other biotic factors also drive variation in disease symptoms, particularly disease complexes.

In this study, we aim to overcome some of these limitations by employing a quantitative reduced representation sequencing (qRRS) assay to simultaneously measure the abundance levels of pests of interest and the associated metagenomic community. This approach offers similar sensitivity and precision as shotgun metagenome sequencing but at a low cost [17]. Consequently, it is amenable to metagenomic studies at a population-level scale. The qRRS method offers a taxonomically comprehensive profile and a more holistic approach to understanding disease outcomes as a product of plant–pathogen–microbe interactions [17]. This study focuses on the plant–insect interactions within sweetpotato leaf metagenomes of biparental and diversity populations. The aims include (i) identifying community members that modulate plant–pest interactions, (ii) understanding the genetic basis for the sweetpotato–whitefly interaction, and (iii) evaluating the genomic prediction abilities for the abundance of the pests while comparing various parameters (i.e. metagenome as a covariate, level of genetic diversity, allele dosage, and relationship matrices) within the various prediction models. The holobiont-aware analysis is based on the premise that the host and the core set of the metagenome exist as a single evolutionary unit and community members modulate host phenotypic expression. While traditional plant genomic approaches have predominantly focused on the host genome, our study explores the holobiont theory to enhance genomic predictions and accurately identify genetic factors underlying traits.

## Results

### Variant calling

Using high-throughput quantitative reduced representation sequencing (qRRS), we obtained genome-wide data of sweetpotato individuals and component genomes of their leaf-associated metagenomic community members. The median, mean, maximum, and minimum read depth in the biparental population are 128, 118, 280, and 45, respectively, while they are 132, 107, 446, and 45, respectively, in the diversity population. The median, mean, maximum, and minimum mapping rates in the biparental population are 93.5%, 93.28%, 94.83%, and 90.8%, respectively, while they are 93.89%, 93.54%, 94.51%, and 89.6%, respectively, in the diversity population. A total of 252 977 (File S1) and 127 287 (File S2) variants (SNPs and indels) were used for both GWA and genomic prediction in the diversity and biparental population (49 334 after filtering for segregation distorted loci (SDL); File S3), respectively. The proportion of dosage-based genotypic classes varied from 5% to 42% in the diversity population and from 4% to 29% in the biparental population before SDL filtering and 3% to 42% after SDL filtering (Fig. S1). While nulliplex markers account for the highest proportion of markers in the diversity population (nulliplex: 42%), simplex markers accounted for the highest proportion of markers in the biparental population (simplex: 29% when SDL are included and 42% when SDL are excluded). The distribution and density of markers across chromosomes had similar trends between the two populations (Fig. S1).

### Host–insect–microbiome interactions

**Figure 1:**
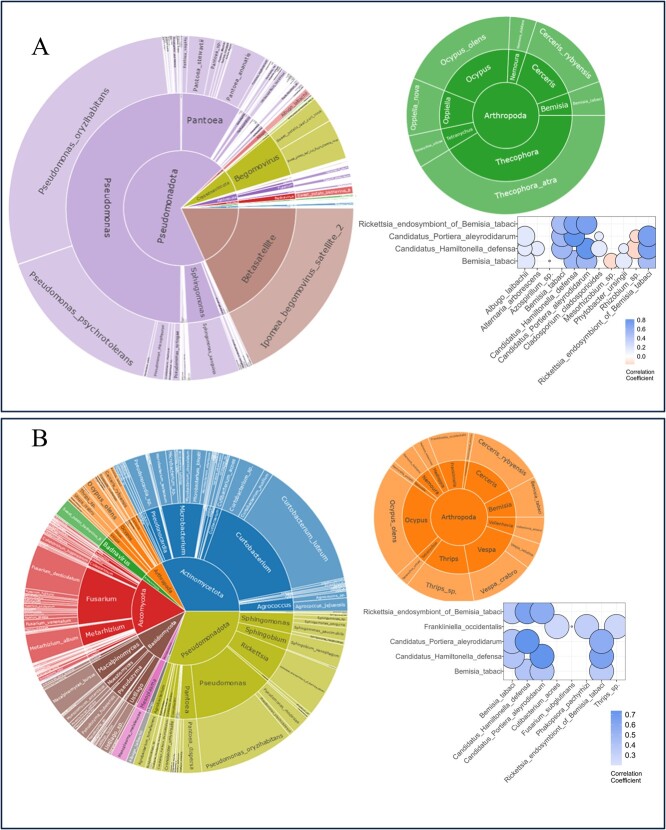
Sunbursts showing the species-level relative abundance of taxa in the leaf-associated metagenomes of the diversity (A) and DC biparental (B) populations (from center to outer ring: phylum, genus, and species). For each population, taxa within the phylum Arthropoda (insects and mites) are shown in the top right sunbursts of each panel, and a subset of the correlation network is shown to highlight significant interactions with *Bemisia tabaci*.

The metagenome is comprised of taxa across a broad range of taxonomic groups spanning viruses to eukaryotes (Files S4-S5). This study focuses on insect species (*B. tabaci, F. occidentalis*, and *O. olens*) that are well represented in each population (Fig. 1). The leaf metagenome profiles were obtained for 767 accessions (File S4) and 454 F1 progenies (File S5) from the USDA germplasm representing global diversity and the biparental population, respectively. *B. tabaci* and *O. olens* were found in a total of 153 and 318 accessions in the diversity population, respectively, while *B. tabaci, F. occidentalis*, and *O. olens* were found in a total of 304, 315, and 340 F1 progenies in the biparental population, respectively.

While the metagenomic profile was resolved down to the strain level, the species level profile identified more taxa and was used for further analysis. In the diversity population, 2503, 4028, and 1732 taxa were identified at strain, species, and genus levels, respectively. In the biparental population, 1622, 2936, and 1304 taxa were identified at strain-, species-, and genus-level, respectively. At the species level, after filtering for taxa that are present in at least 5% of the diversity and biparental populations, a total of 297 and 301 taxa were retained, respectively. Although the metagenome is comprised of more taxa in the diversity population, there are more taxa identified in the biparental population after filtering for taxa that are found in at least 5% of individuals in the population (Fig. 1). While the bacterial taxa dominate (i.e. based on species diversity and relative abundance) the metagenome in the biparental and diversity populations, there were some differences observed in species composition. The phylum Pseudomonadota (majorly *Pseudomonas* spp, followed by *Pantoae* spp, and *Sphiongomonas* spp) quantitatively dominate the diversity population metagenome, followed by viruses. In the biparental population, the phylum Pseudomonadota and Actinomycetota had approximately equal representation of the species abundance, with both dominating the metagenome. In the biparental population, where there is higher bacterial species diversity (Fig. 1), *Pseudomonas* spp. remains one of the two dominant species (including Curtobacterium). The biparental population metagenome also had a higher proportion of fungal and Arthropoda species but viral abundance was much lower in the biparental population.

A total of twenty arthropods, comprising insects and mites, were identified, with notable pests that cause significant yield losses and quality reduction in infested sweetpotato [33]. *B. tabaci*, *F. occidentalis*, *Thrips urticae*, *O. olens*, *Cerceris rybyensis*, and *Thecophora atra* were consistently found across all populations, while the remaining species were specific to one population (Fig. 1). *Bemisia tabaci*, *F. occidentalis* and *T. urticae*, identified from the sweetpotato leaf-associated metagenome, have been previously reported in association with sweetpotato crops [33, 34]. *Ocypus olens*, known for preying on spiders and other small insects, is identified as a predator interacting with sweetpotato [35].

**Figure 2:**
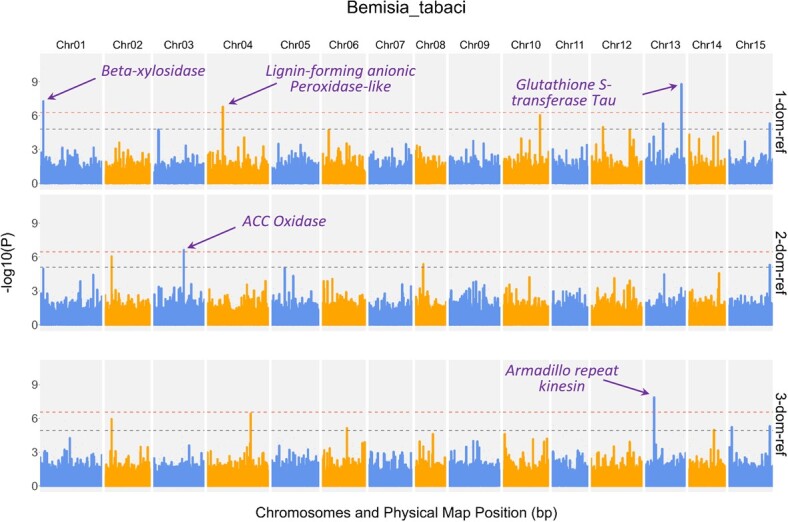
Manhattan plot showing results of dosage-sensitive genome-wide association analysis in the USDA sweetpotato diversity population. The candidate genes underlying the sweetpotato-whitefly interactions are supported by two or more association SNP. The horizontal dashed lines indicate the Bonferroni (upper line) and FDR (lower line) thresholds, respectively.

### Multipartite and multitrophic interactions involving *B. tabaci*

To minimize zero-inflation issues typically associated with metagenome datasets, taxa appearing in less than 5% of samples in a population were eliminated before performing CCLasso-based correlation analyses. As expected in both the diversity and biparental population, moderate and positive correlations were revealed between *B. tabaci* and its endosymbionts, i.e. *Candidatus Hamiltonella defensa*, *Candidatus Portiera aleyrodidarum*, and *Rickettsia endosymbiont of B. tabaci* (Fig. 1A and File S6). The correlations were slightly stronger in the diversity population (Fig. 1; 0.50, 0.54, and 0.54, respectively) than in the biparental population (Fig. 1B and File S7; 0.40, 0.41, and 0.40, respectively). High and positive correlations (0.71–0.81) were found among these endosymbionts but not with other insects and mites. Additionally, *B. tabaci*, *Candidatus Hamiltonella defensa*, and *Candidatus Portiera aleyrodidarum* had significant positive correlations (0.17, 0.16, and 0.17, respectively) with *Albugo laibachii*, an oomycote known to induce host plant susceptibility to parasites by compromising the plant's defense response (Fig. 1; [36]).

A negative correlation was observed with two plant growth-promoting bacteria, *Mesorhizobium* sp. (−0.16) and *Azospirillium* sp*.* (−0.15), and *B. tabaci*. While *B. tabaci* and the virus (sweetpotato leaf curl virus) it transmits were found in the sweetpotato leaf metagenome (Files S6-S7), significant correlations underlying the insect–viral interaction were not found by the CCLasso network correlation analysis. However, a weak correlation (0.13) was observed between *B. tabaci* and sweetpotato leaf curl virus using the Spearman correlation analysis. Although probably indirect interactions, noteworthy are the weak and negative correlations observed between the endosymbionts of *B. tabaci, Candidatus Hamiltonella defensa* (−0.17) and *Candidatus Portiera aleyrodidarum* (−0.15), and *Rhizobium sp.,* a bacterial genus known for promoting plant growth and defense [37]. These metagenomic data and correlations underscore evidence for multipartite and multitrophic interactions between *B. tabaci* and various microbes that influence the host plant's defense response.

**Figure 3:**
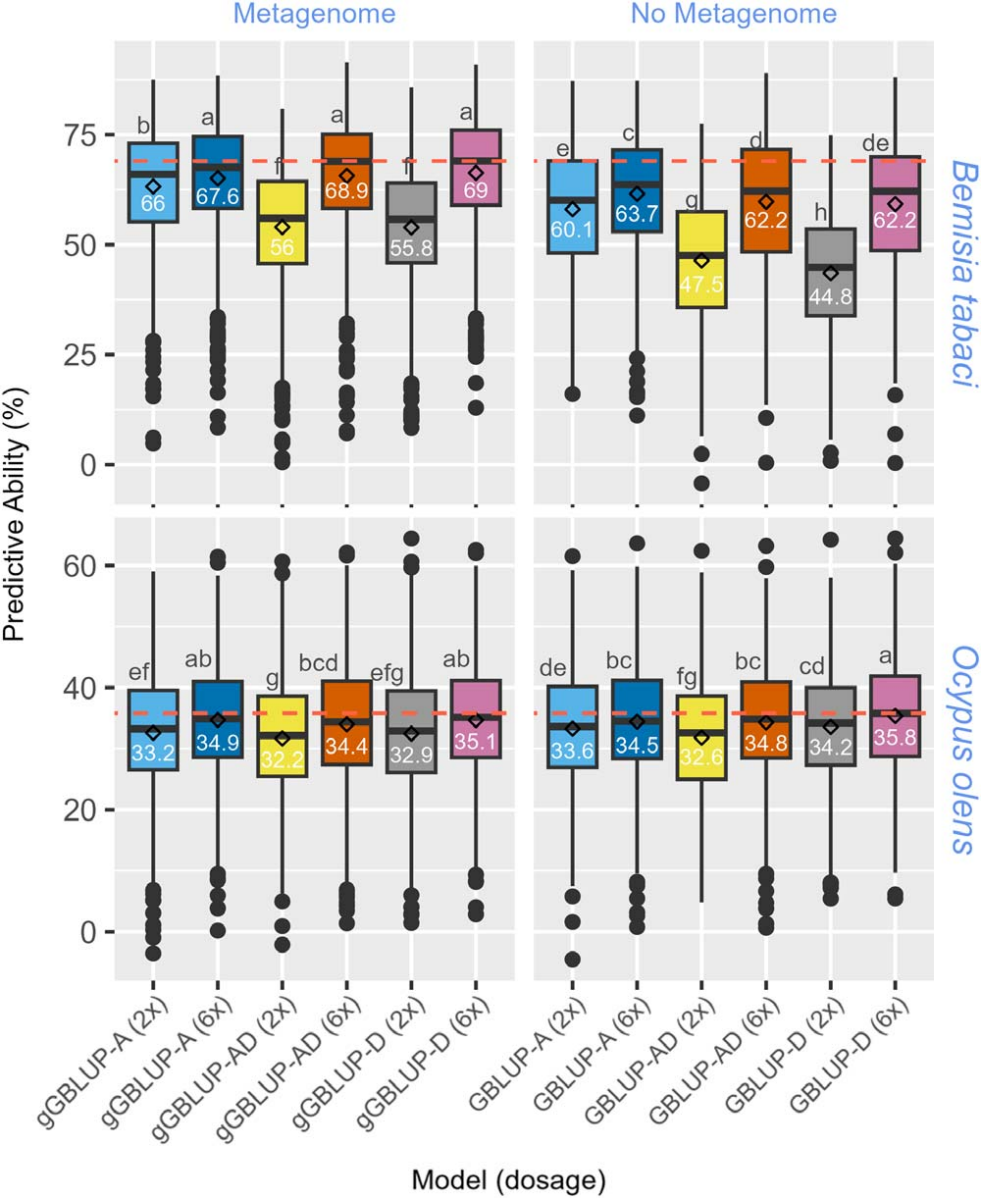
Boxplot showing prediction abilities of abundance of *Bemisia tabaci* and *Ocypus olens* in the sweetpotato USDA diversity population. The means comparison, based on the Duncan multiple range test, shows significant differences between models. The horizontal line within each box indicates the median (value in each box), while the diamonds indicate the mean.

**Figure 4:**
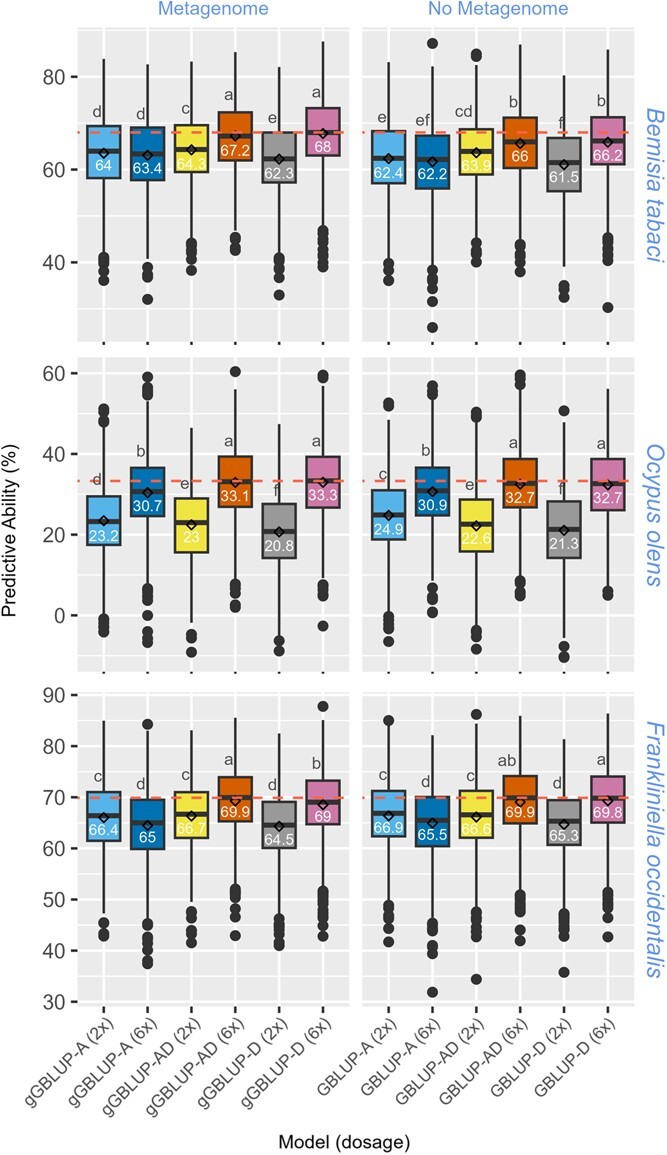
Boxplot showing prediction abilities of the abundance of *Bemisia tabaci, Frankliniella occidentalis,* and *Ocypus olens* in the sweetpotato DC biparental population based on marker data that includes segregation distorted loci. The means comparison, based on the Duncan multiple range test, shows significant differences between models. The horizontal line within each box indicates the median (value in each box), while the diamonds indicate the mean.

### Sweetpotato genetic factors underlying abundance of *B. tabaci*

A genome-wide association analysis (GWA) was performed to understand the genetic basis and identify the candidate genes and pathways that control the host–insect interaction between sweetpotato and *B. tabaci*. To determine if other members of the metagenomic community play a role in the host–insect interaction, GWA was performed with and without the metagenome as a covariate (Fig. S2). The metagenome was used as a covariate in the linear mixed model and observed to reduce false positive rates. Using the stringent Bonferroni threshold, all significantly associated SNPs were identified only in the dominance models (1-dom-ref, 2-dom-ref, and 3-dom-ref), while there were no significantly associated SNP in the additive model at both the FDR and Bonferroni thresholds and regardless of fitting the metagenome as a covariate (Fig. 2). The candidate genes were selected based on their proximity to the most significantly associated SNP (lowest *P* value) and were supported by two or more significantly associated SNPs. The most significantly associated SNPs were all colocalized within gene sequences (Table S1). The QQ-plot was also used to select models that did not suffer from high false discovery rates (Fig. S2). The candidate genes orthologs are involved in plant defense response to herbivory. These genes include ACC oxidase (1-aminocyclopropane-1-carboxylic acid oxidase), β-d-xylosidase, lignin-forming anionic peroxidase, no pollen germination related 1 (NPGR1), and an armadillo repeat kinesin.

### Impact of genetic diversity, metagenome, allele dosage, and relationship matrices on PA

Various GBLUP models (i.e. GBLUP-A, GBLUP-AD, and GBLUP-D) were run with pseudo-diploidized (2×) and 6× dosage genotype datasets. A gBLUP model that used a metagenome-based Cao dissimilarity matrix was also fitted in the mixed linear model for prediction instead of the marker-based GBLUP models. The impact of metagenome as a covariate and the level of genetic diversity (diversity vs. biparental population) was also evaluated across all the models. These analyses were performed to predict the relative abundance levels of three insect species across the diversity (*B. tabaci* and *O. olens*) and biparental (*B. tabaci*, *F. occidentalis* and *O. olens*) populations. The holobiont-aware genomic prediction models (gGBLUP: metagenome-enabled GBLUP), using members of the metagenome that are correlated with the taxa of interest as covariates, aims to capture the role of the metagenome/microbiome in the modulation of host–insect interactions. *B. tabaci* and *F. occidentalis* are sap-sucking herbivores, while *O. olens* is a predator that might indirectly interact with sweetpotato plants.

**Figure 5:**
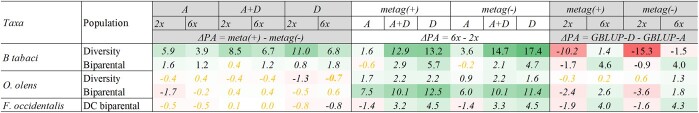
Heatmap showing improvement in predictive ability (PA) based on models with and without metagenome as a covariate, 6× dosage vs. pseudo-diploidized genotypic data, and GBLUP-D versus GBLUP-A. The positive values indicate a gain in PA, while the negative values indicate reduced PA. PA values in the yellow text are not significantly different changes.

**Figure 6:**
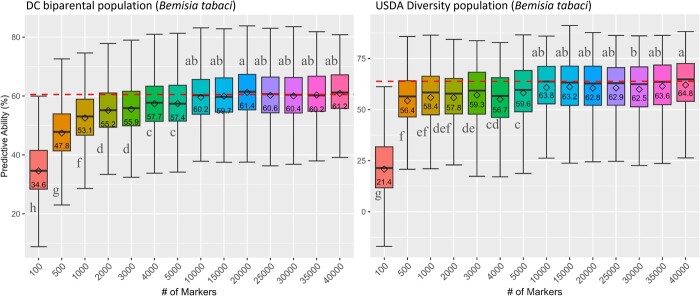
Effect of marker density on prediction ability for the DC bi-parental and the USDA diversity population. The genomic prediction model is based on the GBLUP-A, 6× dose markers, and without metagenome as a covariate. The means comparison, based on the Duncan multiple range test, shows a significant difference between marker densities.

Using the metagenome alone (gBLUP), without the G-matrix, moderate PA were achieved for *B. tabaci* (49.1% and 35.3% in diversity and biparental populations, respectively) and *F. occidentalis* (40.9% in biparental population). The likely indirect interaction between sweetpotato and *O. olens* was marked by low PA of 10.1% and 3% in diversity and biparental populations, respectively. The highest predictive ability in the diversity population was 69% and 35.8% for *B. tabaci* and *O. olens*, compared to 68% and 33.3% in the biparental population, respectively (Figs 3 and 4). The PA based on the metagenome-enabled GBLUP models was significantly higher across all models for *B. tabaci* in both biparental and diversity populations The improvement in PA ranged from 3.9% to 11% in the diversity population and 0.4% to 1.8% in the biparental population. The genomic prediction model for *B tabaci* in the diversity population benefited more from the metagenome as a covariate than in the biparental population. For *F. occidentalis and O. olens*, there was no significant difference in PA across most of the models when assessing the impact of the metagenome as a covariate (Fig. 5).

For comparison between the pseudo-diploidized (2×) and dosage (6×) models, the 6× dosage models consistently outperformed the 2× models across most of the models and taxa evaluated, except for a few cases with the additive model. The gain in PA due to using 6× dosage genotype data was as much as 17.4% (Fig. 5). For comparison between additive and dominance effects, higher PA values were revealed for the GBLUP-D when using 6× dosage data, but the contrary was the case for the 2× pseudo-diploidized data. It should be noted that models that use the 2× pseudo-diploidized models often produced lower PA, particularly when modeling dominance effects (Figs 3–5). It should also be noted that re-coding the 6× dosage data as 2× pseudo-diploidized genotype leads to loss of dosage information, adding to lower PA when modeling dominance effects in polyploids. In the biparental population, the impact of filtering segregation distorted loci (SDL) on PA was evaluated. PA was higher for all models when SDL was included in the genotypic data used for computing the G-matrix. Gain in PA due to including SDL ranged from 6.4% to 10.4%, 9.8 to 17.1, and 6% to 9.6% for *B. tabaci*, *O. olens*, and *F. occidentalis*, respectively (with SDL: Fig. 3; without SDL: Fig. S3). To improve confidence in candidate gene selection, we performed local LD analyses for each significantly associated variant. The region around the associated variant on Chr13, based on the 1-dom-ref model, had relatively higher LD spanning multiple possible genes (Figs S4–S8).

### Impact of marker density on PA

To determine optimal marker density for genomic prediction in sweetpotato, PA was estimated based on marker densities ranging from 100 to 40 000 markers in both the diversity and biparental population (Fig. 6). The analysis was limited to the GBLUP-A model, 6× dosage data, and without metagenome as a covariate. A similar trend was found in both populations as PA increased with increasing marker density until it plateaued at 10 000 markers. There was no significant difference between marker density from 10 000 to 40 000 markers. The optimal marker density at 10 000 markers provided 3% to 6.7% more PA than the typical 2000- to 5000-marker density typically used in most genomic prediction studies.

## Discussion

In this study, we demonstrate the implementation of metagenome-enabled genome-wide association analysis (GWA), metagenome-wide association analysis (MWAS), and genomic prediction in hexaploid sweetpotato. We utilize genome-wide data along with species-level metagenomic profiles obtained simultaneously from a Next-Generation Sequencing (NGS) based assay (qRRS).The background method, without the G-matrix, was also used for prediction. Plant–pest interactions were evaluated by estimating the abundance of insects from the relative abundance of DNA present in the metagenome. We present key findings as follows (i) based on correlation analysis of the compositional data, species-level profiling found multipartite and multitrophic interactions, some of which corroborate known interactions. These correlations identified biologically relevant organismal interactions that underscore host–metagenome co-evolution and modulate seemingly unrelated traits, e.g. whitefly-rhizobia antagonism might be relevant to herbivory resistance based on the nitrogen status in the form of nitric oxide [38, 39]; (ii) candidate genes identified from GWA implicate the ethylene-dependent plant defense response pathway and cell wall modification as important for resistance to the sap-sucking whitefly; (iii) holobiont-aware models reduce GWA false-positive rates and can improve PA; (iv) modeling allele dosage (6×) and digenic dominance G-matrix perform better for prediction than pseudo-diploidized genotypes and additive relationship matrix, respectively; and (v) optimal PA was achieved at a marker density of 10 000 in both the diversity and biparental populations.

The correlation network analysis enabled the identification of endosymbionts directly or indirectly interacting with host plants. Positive correlations with insects suggest potential synergistic interactions. Bacterial symbionts have been shown to play key roles in insect reproduction, immunity, and nutrition [40]. These interactions can extend to multiway interactions; for example, Zhao *et al*. [41] revealed that viruses can manipulate and reprogram plant immunity to improve the performance of the insect vector but suppress the performance of nonvector insect herbivores. Specific interactions can have unknown far-reaching global consequences that drive other important host–microbe interactions.

One of the major drawbacks of GWAS is that the analysis often identifies spurious associations even after controlling population structure. Likewise, due to multipartite interactions, other biotic factors can lead to spurious associations. We posit that accounting for these multipartite and multitrophic interactions can significantly reduce spurious associations. For example, several studies have revealed antagonism between the jasmonic acid (JA) and salicylic acid (SA) pathways, so that the host becomes more susceptible to a group of organisms that are limited by the downregulated defense response pathway [42]. Implementation of a metagenome-enabled GWA reduced false-positive rates in this study, as shown in the QQ-plot and Manhattan plots. Significantly fewer and well-defined regions of the genome retained associations after dropping spurious hits. This resulted in candidate genes that are more functionally relevant to the trait of interest. We successfully pinpointed herbivory-limiting candidate genes that were supported by multiple SNPs and based on the stringent Bonferroni threshold. These candidate genes were only found in the dominance models, downplaying the role of additive effects in sweetpotato–whitefly interaction.

It is worth noting that association analyses (QTL or GWAS) for resistance to sweetpotato insect pests are limited as most sweetpotato disease studies have primarily focused on microbial pathogens. Our study addresses this gap by providing a practical and straightforward approach to estimating the level of pest infestation. ACC oxidase plays a pivotal role in ethylene biosynthesis, a hormone linked to plant stress responses. Research has substantiated that ACC oxidase proteins play a crucial role in enabling plants to respond to biotic stress by up-regulating the production of ethylene [43]. Studies have also suggested an intricate interplay between ET) and JA through the ET/JA-mediated signaling pathways, which co-regulates the expression of genes involved in plant defense [11]. β-d-xylosidase is an enzyme that plays a crucial role in the breakdown of hemicellulose, a component of plant cell walls. This corroborates knowledge about cell wall thickness and lignification as the basis for the first line of defense against feeding by herbivores [44]. The lignin-forming anionic peroxidase catalyzes the formation of lignin polymers by forming rigid cross-links between lignin, cellulose, and extension in the secondary plant cell wall. The observed association underscores the importance of lignin in plant defense mechanisms by reinforcing cell walls and deterring herbivore feeding. The Glutathione S-tansferase Tau gene plays critical roles in detoxification, stress tolerance, and antioxidative defense in plants [45]. Their involvement in plant–pathogen interactions Armadillo repeat kinesin genes, versatile anterograde transporters in plants, are believed to play roles in intracellular transport and transport of organelles [46]. Chloroplast movement, positioning, and accumulation at the site of infection have been described as an effective defense response mechanism by providing pathogen penetration resistance or acting as the main source of ROS formation [47].

Following the evaluation of various parameters across genomic prediction models, using the diversity population, metagenome as a covariate, 6×-dose genotypes, and the dominance model were superior to their alternative options. While the expectation is that predictive abilities will be higher in a genetically less diverse biparental population, our results consistently show similar PA. It is expected that allele dosage will be critical for genomic prediction in polyploids, particularly for traits where dominance effects are important. While GBLUP-D produces higher PA than GBLUP-A, the exception was the case for models based on pseudo-diploidized 2× data. This is expected since a significant amount of dosage information is lost during pseudo-diploidization of the 6×-dose genotypes. Consequently, this does not indicate that the GBLUP-A outperforms GBLUP-D, rather, the pseudo-diploidized 2× genotype data lacks the dosage information required to accurately model the dominance relationship matrix. The G-matrix used for genomic prediction often models additive genetic effects due to the challenges of incorporating dominance effects as a result of factors, such as a high allele dropout rate. It is even more challenging in polyploids where the number of alleles required to produce a dominant phenotype is variable (i.e. 1–5 doses in hexaploid sweetpotato). The allele dose-sensitive sequencing (qRRS), dosage-based variant calling pipeline, and the model by Slater et al. [29] have significantly improved our ability to capture dominance effects in the G-matrix. This is particularly important for breeding in polyploid crops. When dominance plays a significant role in trait expression, modeling dominance effects is desired since superior trait values are maintained in a higher proportion in a population. This is particularly true when a single dose of the dominant allele is required to express the dominant phenotype.

As expected, higher PA were observed in *B. tabaci* and *F. occidentalis* that form close interactions with the sweetpotato host. The PA for GBLUP, gGBLUP, and gBLUP of *O. olens* were consistently lower in both populations, indicating that this is an indirect interaction with the host plant. Studies on oviposition behavior revealed that *B. tabaci* and *F. occidentalis* insert their eggs into plant leaf tissue, while *O. olens* don’t. Considering the size of *O. olens* and the limited interaction with living plant tissue (interaction based on predation of plant pests), *O. olens* is unlikely to leave a significant amount of its DNA behind on the plant leaf. It is possible the BLAST match of the metagenome reads is identifying a species closely related to *O. olens* but whose sequences are absent in the NCBI database. Besides improving genomic predictive ability, the modeling of the background metagenome alone (i.e. without the G-matrix) for predicting relative abundance highlights its key role in modulating phenotypic expression.

## Conclusion

Our findings confirm the holobiont theory of host–metagenome co-evolution and that the concept can be applied to significantly improve the accuracy of genomic estimated breeding values. Using only variation in the host genome for genomic prediction, predictive accuracy is typically limited by missing heritability problems. Therefore, we propose that considering the variation attributed to the host-associated metagenome could effectively mitigate certain aspects of the missing heritability problem.There is growing interest in the implementation of metagenome-enabled genomic prediction [48]. While some traits may be perturbed to a lesser degree by the metagenome, it is expected that the approach will be amenable to agronomic traits such as disease resistance, stress tolerance, nutrient acquisition in plants, feed efficiency, obesity in animals, and methane production in ruminants.

## Materials and methods

### Plant materials

A USDA diversity population representing global genetic diversity, composed of 767 sweetpotato accessions, and a DC biparental population, composed of 454 sweetpotato F_1_ progenies, were used to determine the impact of high and low genetic diversity on genomic prediction. The diversity population was also used to perform a genome-wide association analysis. The diversity population accessions were obtained as tissue culture-derived plantlets from the USDA, ARS PGRCU germplasm repository (Griffin, GA, USA) and then maintained in a greenhouse at the USDA-ARS Vegetable Laboratory (Charleston, SC, USA). The biparental population was derived from a cross between DM04-001 (also known as NCDM04-001) and Covington (DC biparental population). The DC biparental population was obtained from germinated seeds and maintained in a greenhouse at NC State University (Raleigh, NC, USA). The parents, DM04-001 and Covington, of the DC biparental population have contrasting characters. DM04-001 has high dry matter, purple-reddish skin, and light-yellow flesh, while Covington has low dry matter, rose skin, and orange flesh high in β-carotene.

### DNA extraction, qRRS library preparation, and sequencing

Young and fully expanded sweetpotato leaves were sampled from the greenhouse plants and used for total genomic DNA extraction. The lyophilized leaf tissue samples of the diversity population were subjected to DNA extraction using a DNeasy Plant Mini Kit (Qiagen), while fresh leaf samples of the biparental population were frozen in liquid nitrogen and subjected to DNA extraction based on a modified CTAB-based protocol [18]. DNA purity and concentration were assessed with NanoDrop 2000 spectrophotometer (ThermoFisher). The samples were then quantified with the Invitrogen Quant-iT™ PicoGreen™ dsDNA Assay and normalized to 100 ng/ul. A GBSpoly NGS library preparation (a previous version of the library preparation method now termed OmeSeq-qRRS following protocol modifications) was previously described by Wadl et al. [19]. The library preparation and sequencing were performed to simultaneously obtain sequences for variant calling and metagenome profiling. The normalized DNA samples were double digested with *Cvi*AII and *Tse*I and then ligated to barcoded adapters with 6 to 9 bp variable length barcodes downstream of 6-bp buffer sequences. The sequence composition ensures that the restriction recognition sites are not reconstituted. The pooled samples were double-digested with the same enzymes (*Cvi*AII and *Tse*I) to remove chimeric fragments. Fragments were size selected for 300 to 600 bp fragments using the Blue Pippin Prep System (Sage Science), enriched with 18 cycles of PCR (NEB Phusion high-fidelity polymerase, New England Biolabs), size-selected again for 300 to 600 bp fragments, and sequenced on the Illumina HiSeq 2500 system.

### Demultiplexing, quality filtering, variant calling, and metagenome profiling

As previously described [19], the Illumina NGS fastq data were demultiplexed and quality filtered using the ngsComposer pipeline [20]. The reads were consequently used for variant calling using the GBSapp pipeline (https://github.com/bodeolukolu/GBSapp). The dosage-based variant calling of the hexaploid (6×) sweetpotato and variant filtering parameters were previously described by Wadl et al. [19]. The reads were aligned against the diploid *Ipomoea trifida* and *Ipomoea triloba* reference genome assemblies (http://sweetpotato.uga.edu/; [21]). Only variants derived from conserved sequences mapping to both genomes were used for downstream analysis. Since sweetpotato is an allo-autopolyploid, the GBSapp variant calling and filtering pipeline uses the closest putative ancestral diploid progenitor (*I. trifida*) and the most distantly related diploid within the batatas species complex (*I. triloba*) as reference genomes. Consequently, conserved sequences between *I. trifida* and *I. triloba* genomes are expected to be likely found in other crop wild relatives and the hexaploid sweetpotato genomes. Consequently, the dosage would be expected to be 6× dose (6 haplotypes). The SNPs were anchored to the *I. trifida* reference genome. Following variant filtering, 252 977 (File S1) and 127 287 (File S2) variants were used for downstream analyses in the diversity and biparental populations, respectively. After filtering to exclude segregation-distorted loci in the biparental population, a total of 49 334 variants were kept (File S3). The variant filtering parameters include a read depth threshold of 45 at each genotype call, a minor allele frequency (maf) threshold of 0.05, and no more than 20% missing data across variants and samples.

Using the same fastq files, metagenome profiling was performed with the Qmatey pipeline (https://github.com/bodeolukolu/Qmatey; [17]). The metagenome analysis in the Qmatey pipeline involved an initial removal of host-derived sequences (about 98% of reads that matched the diploid reference genomes). The reads that did not map to the *I. trifida* reference genome were then used for taxonomic identification and quantitative profiling using the NCBI nt database. Details on the Qmatey metagenome analytical workflow are described in Adams et al. [17].

### Holobiont-aware genome-wide association analysis

Genome-wide association analysis (GWA) was performed with the GWASpoly R package [22], using various dosage models (additive, 1-dom-ref, 1-dom-alt, 2-dom-ref, 2-dom-alt, 3-dom-ref, and 3-dom-alt models). The trait data were derived from the relative abundance of *B. tabaci*, which was obtained from the metagenomic profiles. The additive genomic relationship matrix, based on the 6× dosage variant data, was used to account for population structure and computed based on the VanRaden method [23] in the AGHmatrix R-package [24]. To perform a metagenome-enabled GWA, a CCLasso-based correlation analysis (correlation inference for compositional data through Lasso; [25]) was performed and a correlation coefficient threshold of ±0.1 was used to determine taxa that should be included in the model. To minimize the impact of high zero-inflated values typical of metagenomic datasets, relative abundance values of zero for *B. tabaci* were considered missing values, and associated samples with zero values were excluded. Consequently, 153 sweetpotato samples were retained for GWA. In the subsetted sample set, zero values were kept for all other taxa (i.e. members of the metagenome that are correlated with *B. tabaci*). The correlated taxa (a subset of the metagenome) were used as covariates by performing a principal component analysis (PCA) and then the first three principal components were fitted as fixed effects in the GWAS linear mixed model, which is specified as:

\begin{align*} y= W\alpha +X\beta+ Z\textrm{u}+\epsilon, \end{align*}

where α is a c-vector of specified covariates (fixed effects) corresponding coefficients including the intercept; β = vector consisting of fixed effects of each SNP being tested; u = vector of the random additive genetic background effects associated with the lines; e = vector of residual effects, and W, X, and Z are the incidence matrices that relate y to each of α, β, and u, respectively. The relative abundance of taxa used is based on taxa that are correlated with the taxa of interest following a CCLasso-based correlation network analysis implemented within the Qmatey pipeline [17]. The variances of the random effects are modeled as Var (u) = 2KVg, where K = n × n matrix of pairwise kinship coefficients that define the degree of genetic covariance between individuals and Vg = genetic variance [26]. The REML estimates of variance components were estimated following an efficient mixed-model association algorithm method [27, 28], with the optimum compression MLM and the P3D options. This clusters the lines into groups to increase statistical power and computational speed [28].

### Identification and annotation of candidate genes

The candidate gene selection was performed using the *I. trifida* reference genome assembly [21] since the variants were anchored to genomic positions in the *I. trifida* assembly, which spans 462 Mb and contains 32 301 annotated high-confidence gene models (http://sweetpotato.uga.edu/).  *I. trifida* is the closest known ancestral progenitor of hexaploid sweetpotato and the genome has high conservation of synteny with hexaploid sweetpotato. The variants were annotated and anchored to the *I. trifida* reference genome.

### Genomics prediction for sweetpotato-associated insects

The genomic best linear unbiased prediction (GBLUP) method was used for genomic prediction of relative abundance estimates for three insect species (i.e. *B. tabaci, Frankliniella occidentalis,* and *Ocypus olens*) in the diversity and biparental population. An extension of the GBLUP method was also applied by using the background metagenome as a covariate (gGBLUP: metagenome-enabled GBLUP). The relative abundance data of the taxa predicted are excluded from the metagenome data used as a covariate. A metagBLUP (gBLUP) prediction model was also implemented by using the metagenome-based Cao dissimilarity matrix instead of the marker-based genomic relationship matrix (G-matrix) used for GBLUP. While *B. tabaci* and *O. olens* abundance data were used for analysis in both populations, *F. occidentalis* abundance data was only used for analysis in the biparental population. Using the AGHmatrix R-package [24], the additive, dominance, and full autopolyploid (additive and nonadditive) model relationship matrices were computed using the VanRaden, Vitezica, and Slater methods, respectively [23, 29, 30]. For the gBLUP, the Cao dissimilarity matrix was computed with the R-package vegan [31].

The metagenome covariate was modeled as fixed effects (gGBLUP) in the same way described for the GWA model above. After re-coding zero as missing values in the relative abundance of *B. tabaci* and *O. olens* in the 767 samples of the diversity population, 153 and 318 sweetpotato samples were retained for genomic prediction, respectively. After re-coding zero as missing values in the relative abundance of *B. tabaci*, *O. olens*, and *F. occidentalis* in the 454 samples of the biparental population, 304, 340, and 315 sweetpotato samples were retained for genomic prediction, respectively. Genomic prediction was performed using the GAPIT R-package [32] to estimate prediction ability (PA) based on a 5-fold cross-validation method and 1000 iterations (replications) for each of the models. The models are based on GBLUP-A, GBLUP-D, and GBLUP-AD and use the additive, dominance, and full autopolyploid (additive and dominance) relationship matrices, respectively. Predictive abilities were compared among the three models, between two dosage models (2× pseudo-diploidized and 6×), and between models with and without metagenome as a covariate. To test the effect of marker density on PA, 1000 iterations were performed using a GBLUP-A, without metagenome as a covariate, and 5-fold cross-validation. The GBLUP prediction model is described below:

$$ y=1 n\mu + W\alpha + Zu+\in $$

where y = vector of phenotypes (number of phenotypes × 1), 1n = vector of ones, μ = overall mean, W = incidence matrix of metagenomic covariates (when specified and described above in GWAS model), α is a c-vector of specified covariates (fixed effects) corresponding coefficients including the intercept, Z = the known design matrix for genotypes (or background metagenome data in the case of metagBLUP or gBLUP), and u = random. The model assumes $u\sim N\left(0,{\sigma}_a^2 Kor{\sigma}_{a+d+ ad}^2K\right)$ with K = kinship matrix with *a* (additive model), *d* (dominance model), *ad* (full autopolyploid model), or Cao dissimilarity matrix in the case of gBLUP; and that $\in \sim N\left(0,{\sigma}_e^2I\right)$. The PA was computed by performing a Pearson correlation analysis between observed BLUPs and the genomic estimated breeding values (GEBVs). A comparison of model performance was performed using a one-way analysis of variance and visualized with box plots.

## Acknowledgements

We thank Ty Phillips for assistance with planting, maintenance, and tissue sampling of the diversity population. The mention of trade names or commercial products in this article is solely for the purpose of providing specific information and does not imply recommendation or endorsement by the USDA. The USDA is an equal opportunity employer. This study was funded by the USDA-NIFA Hatch/Multistate Project W5157-TEN00539, the Bill and Melinda Gates Foundation (grant ID OPP1052983 and OPP1213329), and the Illumina Agricultural Greater Good Initiative grant.

## Data availability

The NGS data (Illumina short reads) used for the USDA diversity population are available on the NCBI (National Center for Biotechnology Information) SRA database (Bioproject ID: PRJNA880973), while the NGS data for the DC biparental population will be publicly available on the NCBI SRA database.

## Conflict of interest statement

The authors declare no competing interests.

## Supplementary data

Supplementary data are available at *Horticulture Research Journal* online.

## Supplementary Material

Web_Material_uhae135
