# Supplementary material for: Metagenome-enabled models improve genomic predictive ability and identification of herbivory-limiting genes in sweetpotato
Source: Hortic Res. 2024 May 10;11(7):uhae135. doi: 10.1093/hr/uhae135 (PMC11226878; doi:10.1093/hr/uhae135)
Supplement: Web_Material_uhae135 [file web_material_uhae135.zip › Table S1.docx]

**Table S1:** SNPs localized within candidate genes that play a role in the interaction between sweetpotato and whitefly (*Bemisia tabaci*).

| Chromosome position (bp) | Dosage model | Threshold  *(-log_10_P)* | Score  (*-log_10_P)* | Effect | SNP-gene proximity | Candidate Gene orthologs |
| --- | --- | --- | --- | --- | --- | --- |
| Chr01:1218557 | 1-dom-ref | 4.8 | 10.4 | 21.1 | Inside | Beta-D-xylosidase |
| Chr03:14229086 | 2-dom-ref | 5.32 | 7.03 | 14.2 | Inside | ACC oxidase |
| Chr04:4306282 | 1-dom-ref | 4.8 | 6.43 | 16.6 | Inside | Lignin-forming anionic peroxidase |
| Chr13:21696422 | 1-dom-ref | 4.8 | 8.73 | 19.3 | 11.6 Kb | Glutathione S-transferase TAU |
| Chr13:3845278 | 3-dom-ref | 5.3 | 7.63 | -18.0 | Inside | Armadillo repeat kinesin |
