# Supplementary figures and images for: Metagenome-enabled models improve genomic predictive ability and identification of herbivory-limiting genes in sweetpotato
Source: Hortic Res. 2024 May 10;11(7):uhae135. doi: 10.1093/hr/uhae135 (PMC11226878; doi:10.1093/hr/uhae135)

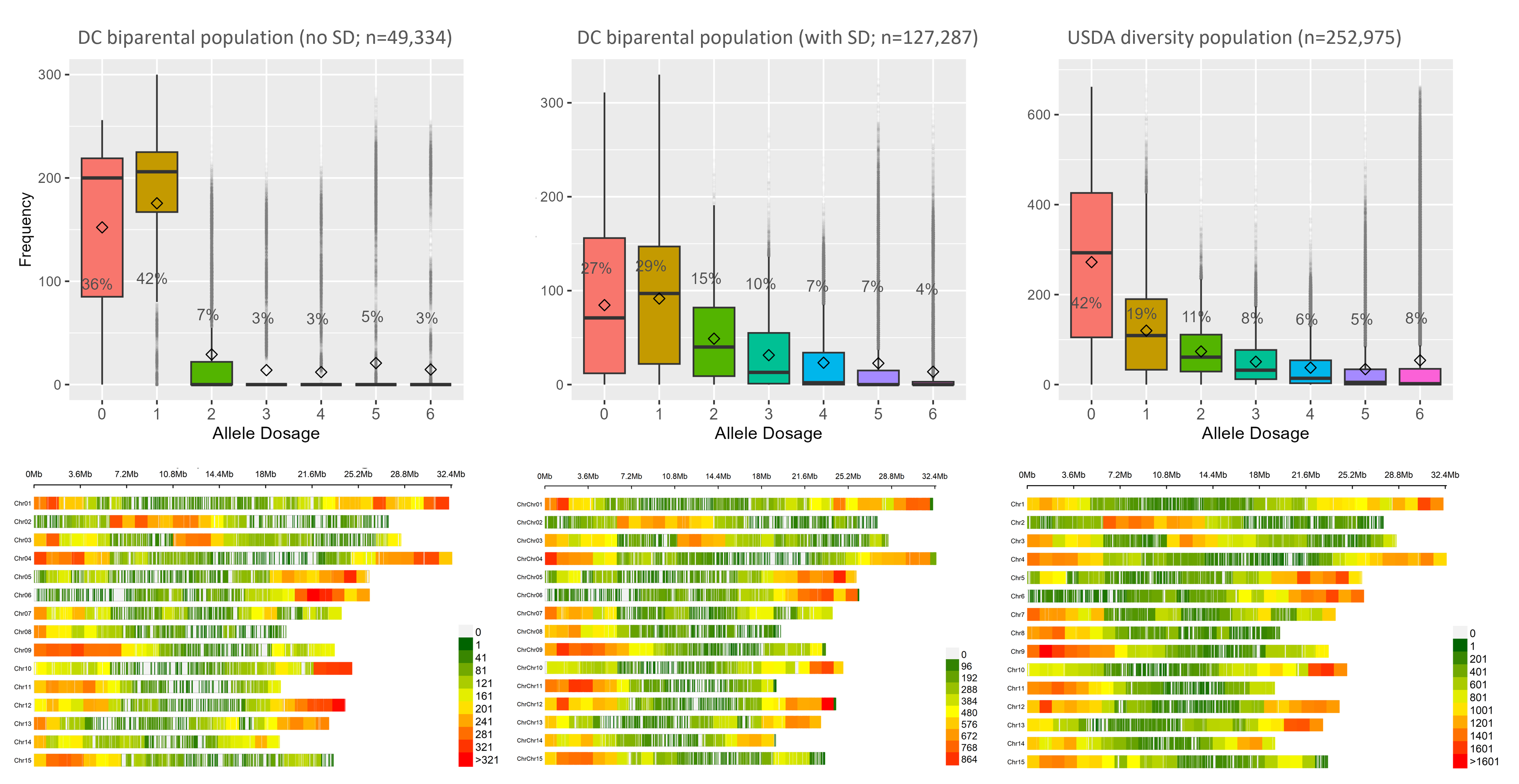

Supplement: Web_Material_uhae135 [file web_material_uhae135.zip › Fig_S1.png]

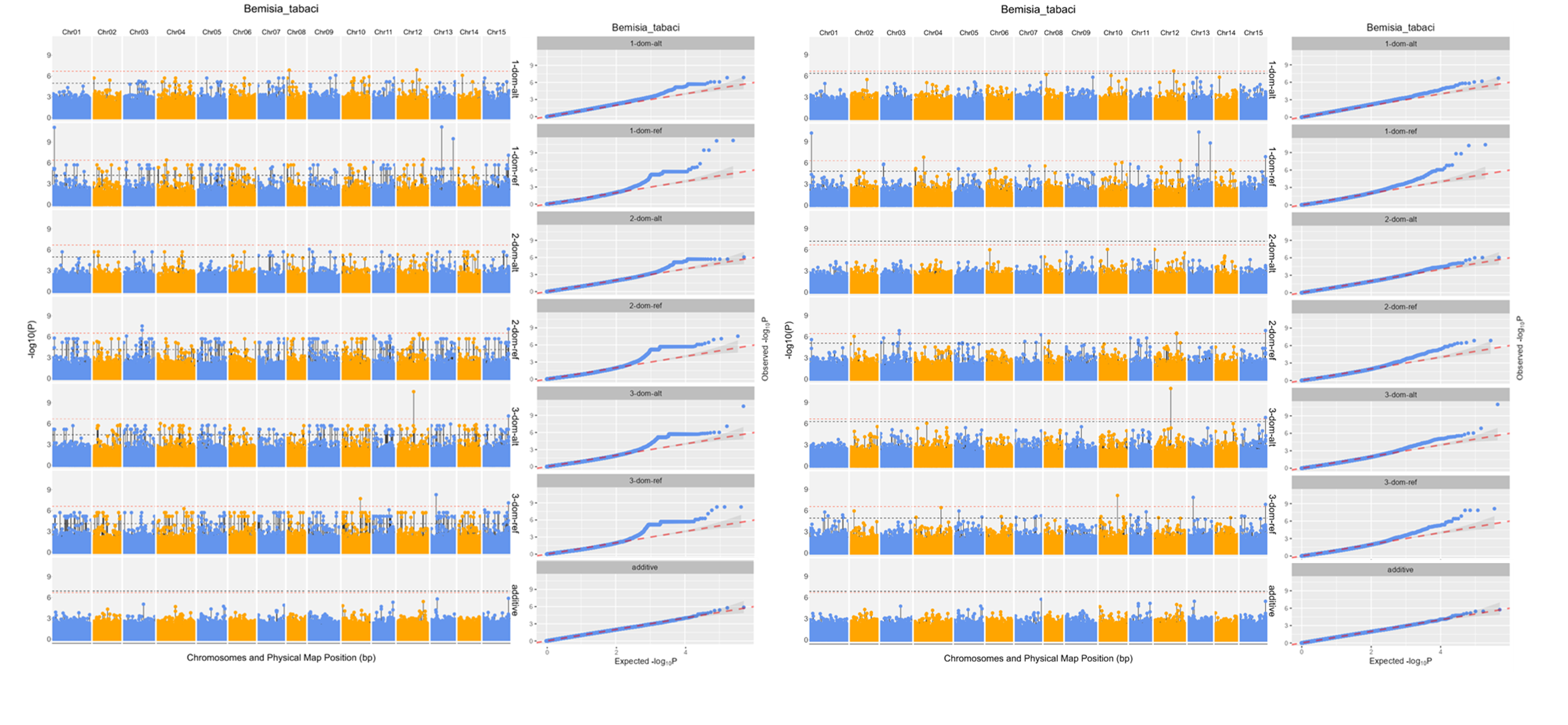

Supplement: Web_Material_uhae135 [file web_material_uhae135.zip › Fig_S2.PNG]

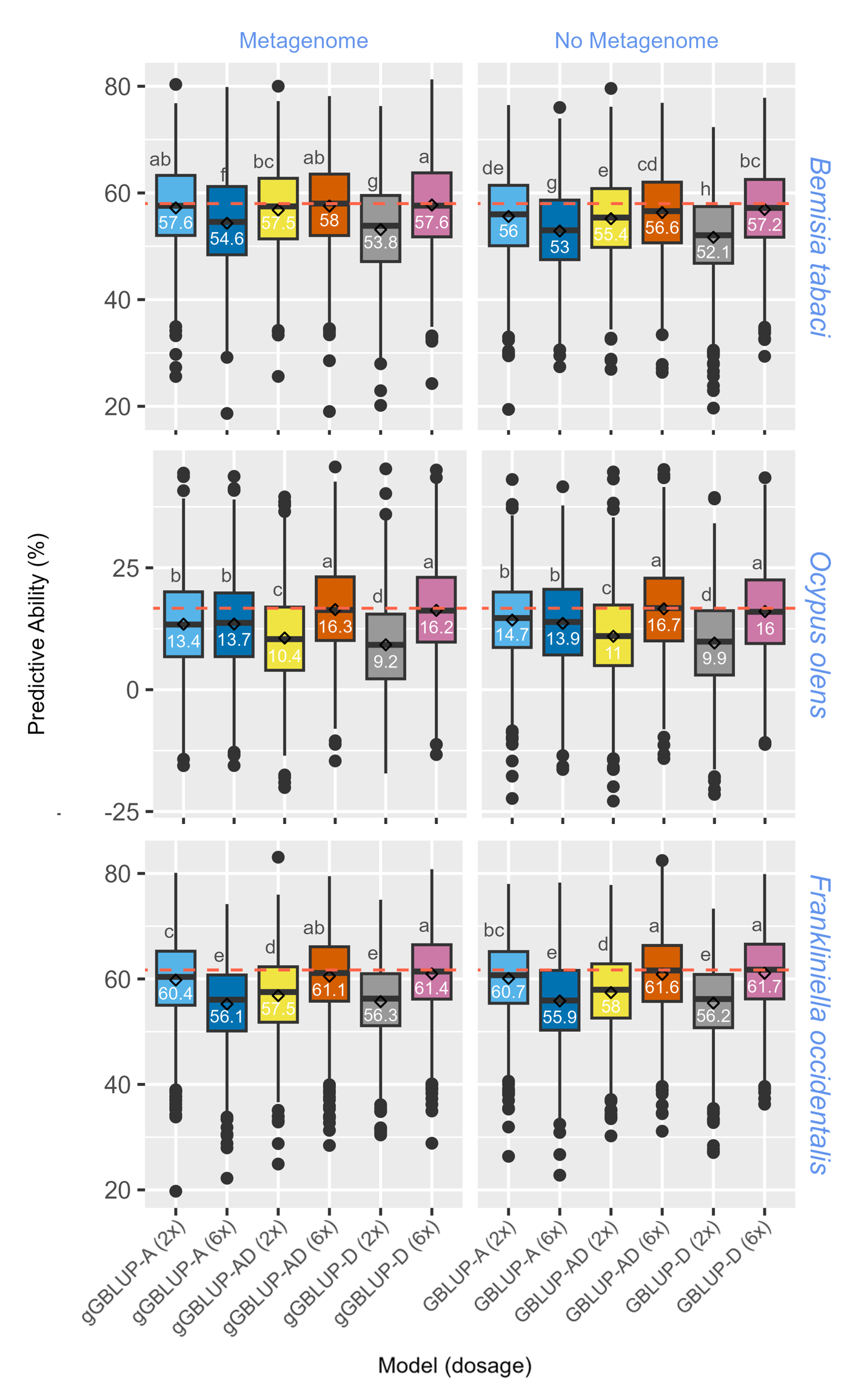

Supplement: Web_Material_uhae135 [file web_material_uhae135.zip › Fig_S3.png]

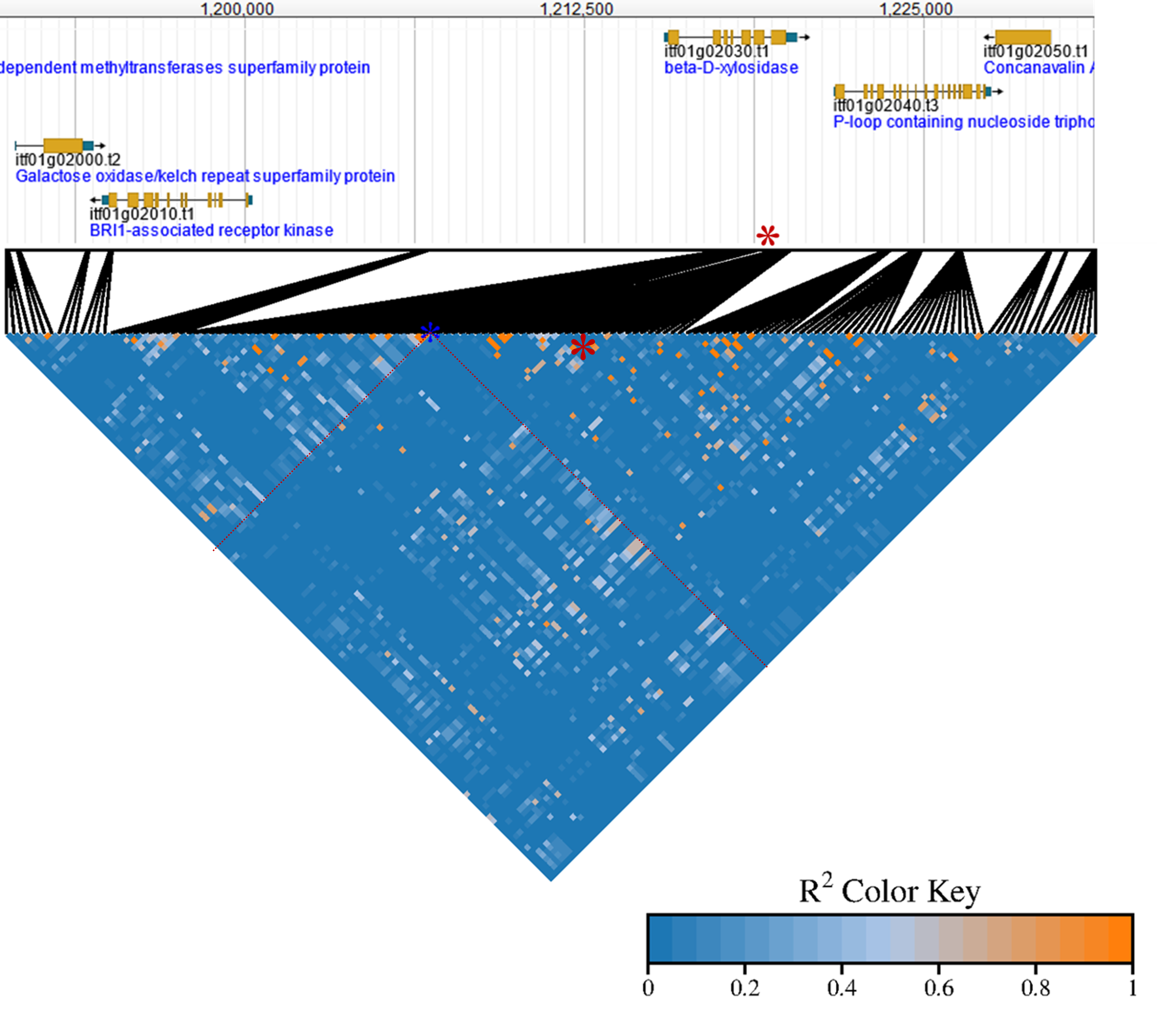

Supplement: Web_Material_uhae135 [file web_material_uhae135.zip › Fig_S4.png]

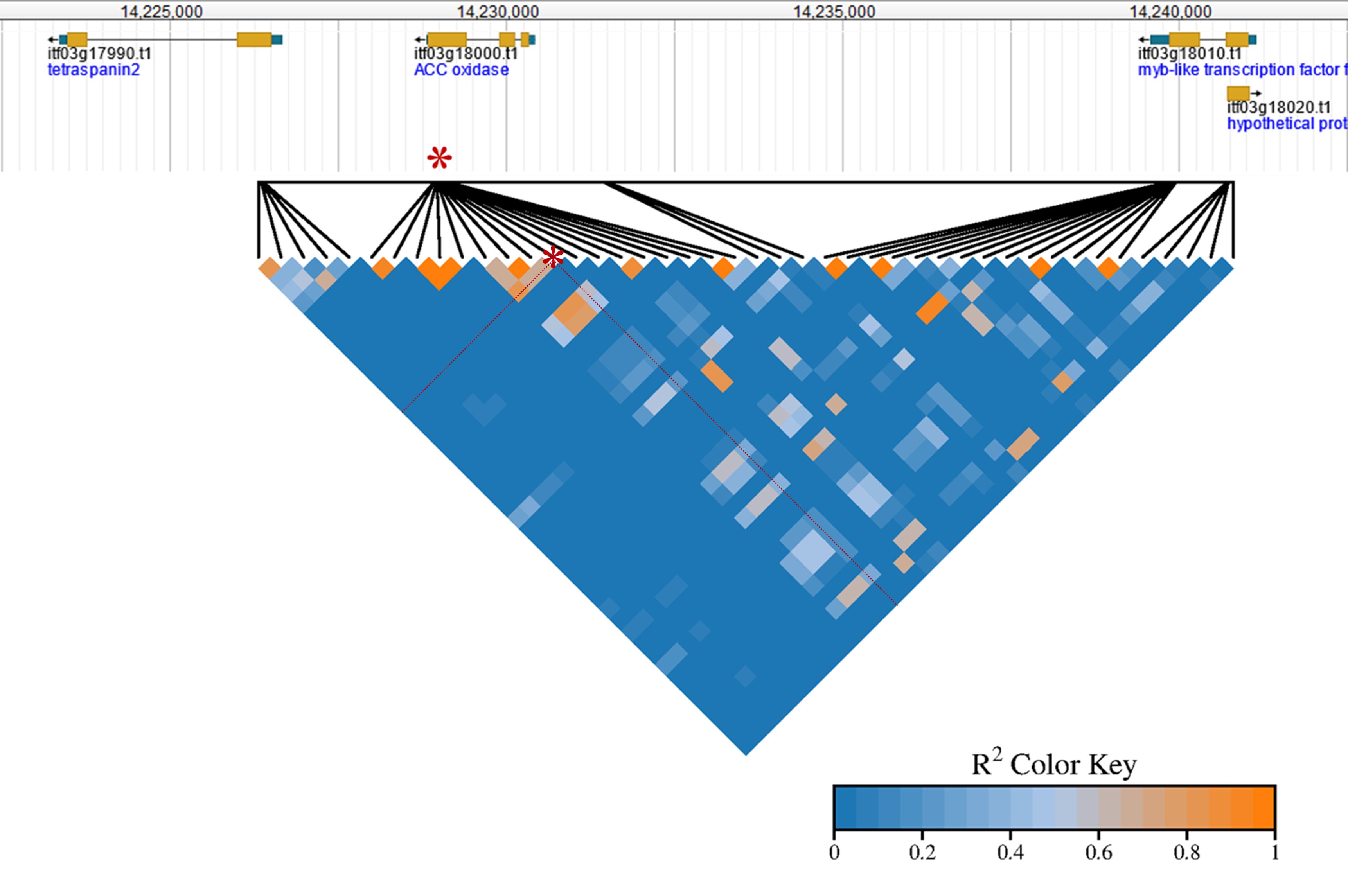

Supplement: Web_Material_uhae135 [file web_material_uhae135.zip › Fig_S5.png]

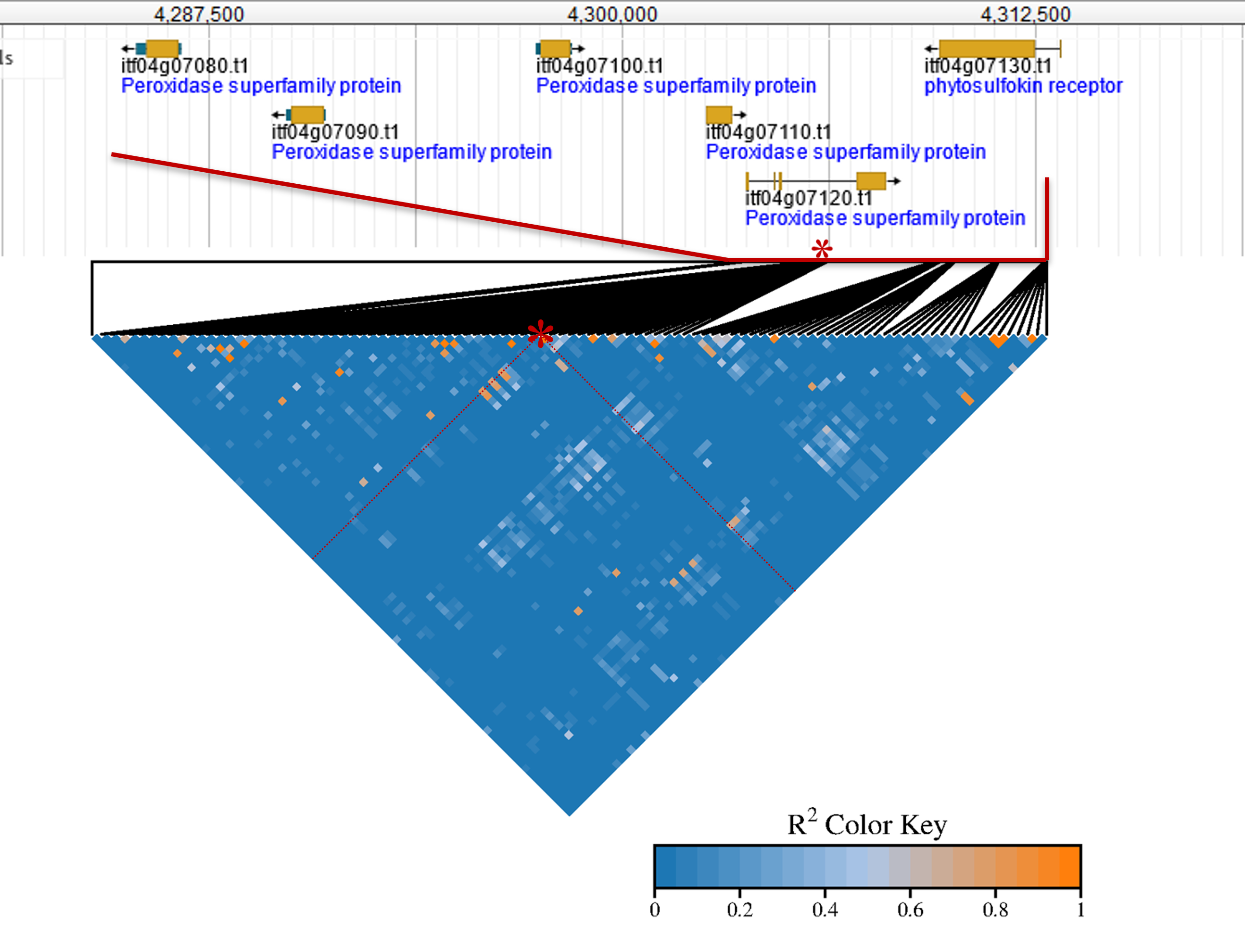

Supplement: Web_Material_uhae135 [file web_material_uhae135.zip › Fig_S6.png]

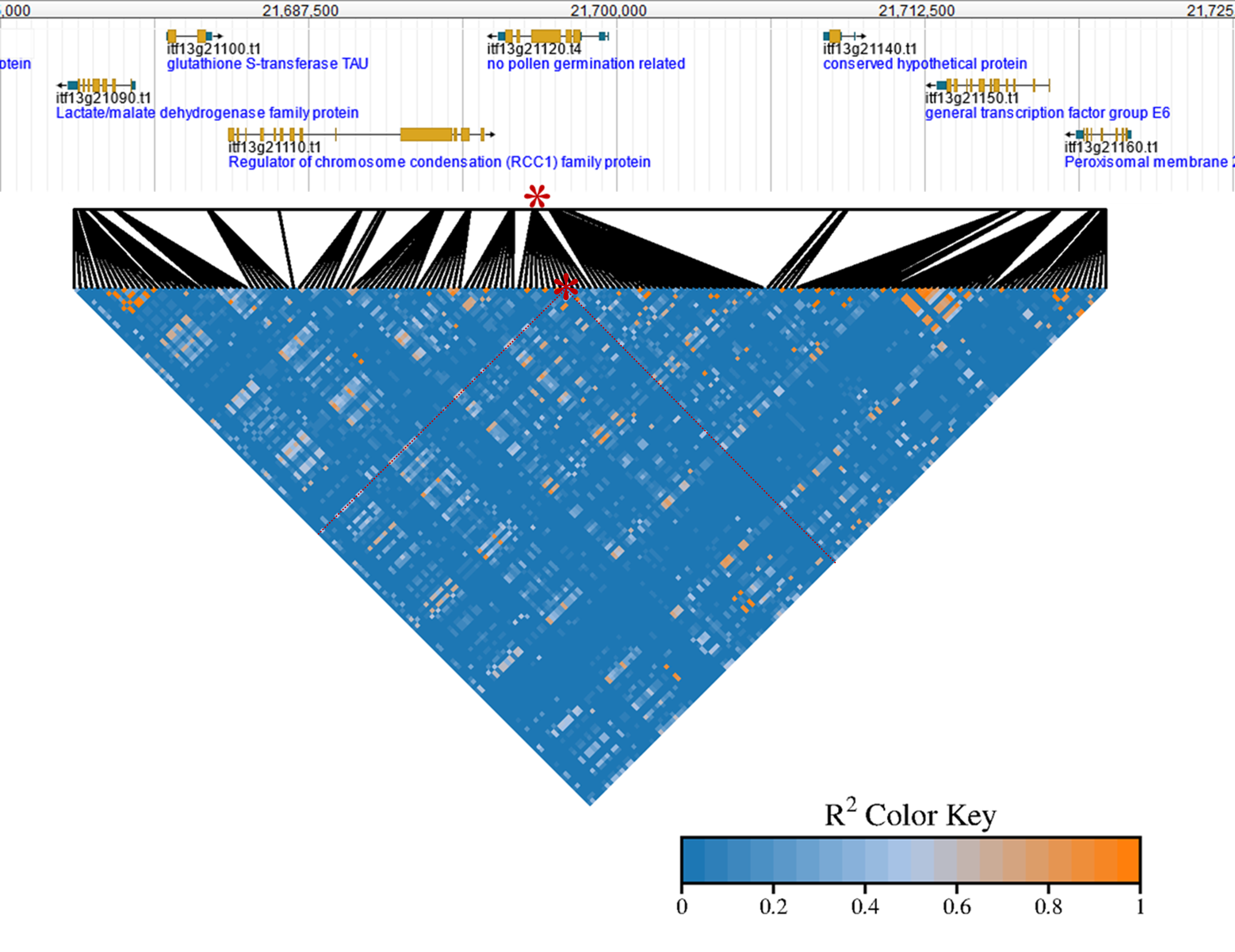

Supplement: Web_Material_uhae135 [file web_material_uhae135.zip › Fig_S7.png]

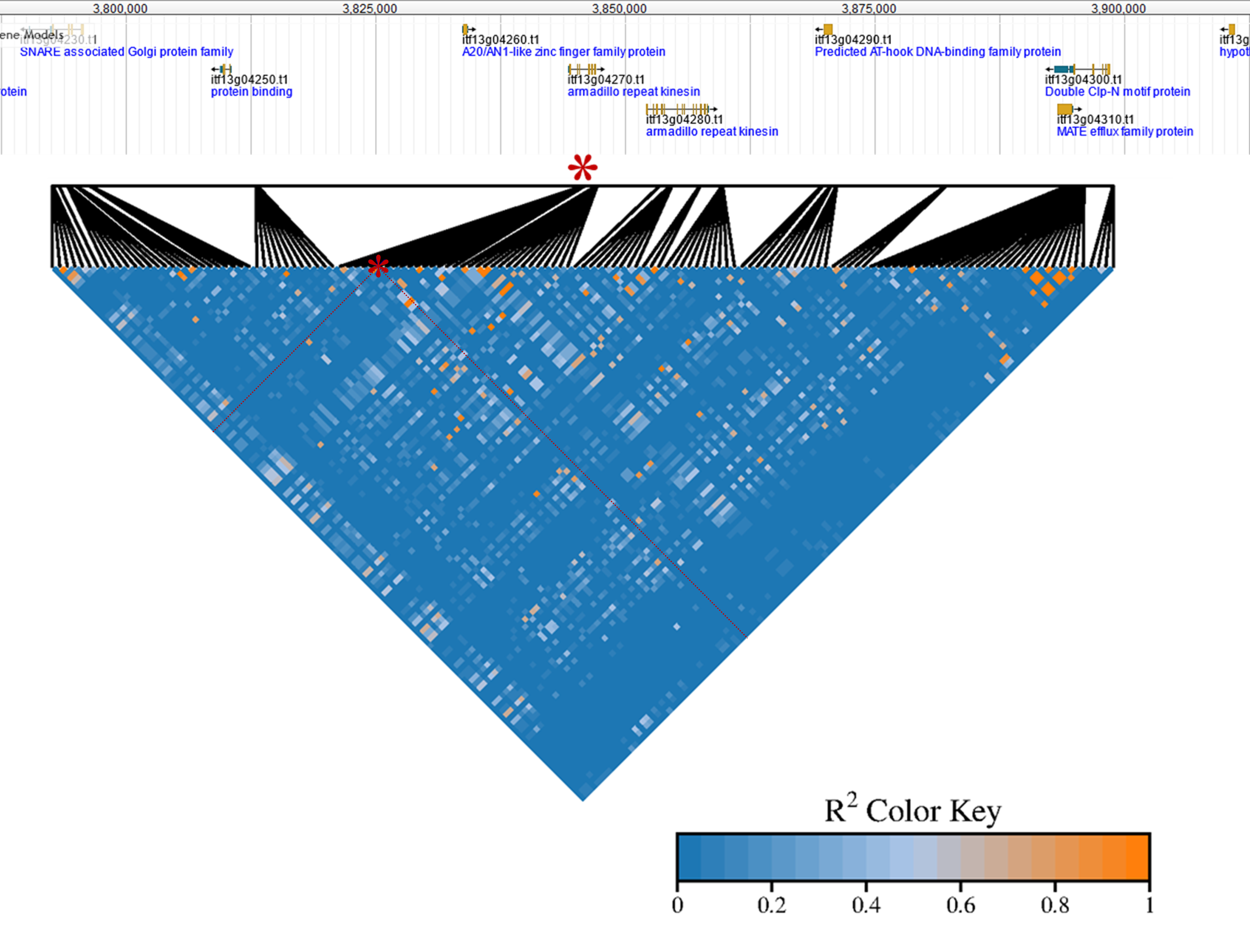

Supplement: Web_Material_uhae135 [file web_material_uhae135.zip › Fig_S8.png]
